# GroPBS: Fast Solver for Implicit Electrostatics of Biomolecules

**DOI:** 10.3389/fbioe.2015.00186

**Published:** 2015-11-17

**Authors:** Franziska Bertelshofer, Liping Sun, Günther Greiner, Rainer A. Böckmann

**Affiliations:** ^1^Computer Graphics Group, Department of Computer Science, University Erlangen-Nürnberg, Erlangen, Germany; ^2^Computational Biology, Department of Biology, University Erlangen-Nürnberg, Erlangen, Germany

**Keywords:** electrostatics, Poisson–Boltzmann equation, finite-difference method, molecular surface, membranes

## Abstract

Knowledge about the electrostatic potential on the surface of biomolecules or biomembranes under physiological conditions is an important step in the attempt to characterize the physico-chemical properties of these molecules and, in particular, also their interactions with each other. Additionally, knowledge about solution electrostatics may also guide the design of molecules with specified properties. However, explicit water models come at a high computational cost, rendering them unsuitable for large design studies or for docking purposes. Implicit models with the water phase treated as a continuum require the numerical solution of the Poisson–Boltzmann equation (PBE). Here, we present a new flexible program for the numerical solution of the PBE, allowing for different geometries, and the explicit and implicit inclusion of membranes. It involves a discretization of space and the computation of the molecular surface. The PBE is solved using finite differences, the resulting set of equations is solved using a Gauss–Seidel method. It is shown for the example of the sucrose transporter ScrY that the implicit inclusion of a surrounding membrane has a strong effect also on the electrostatics within the pore region and, thus, needs to be carefully considered, e.g., in design studies on membrane proteins.

## 1 Introduction

Electrostatic interactions govern the physical–chemical interactions in and between biomolecules (Perutz, 1978). A quantitative description of electrostatic energies (Warshel et al., 2006) is required both for a thorough understanding of biomolecular systems, e.g., of membrane-embedded ion channels or of pK_a_ changes during enzymatic function and in protein design, e.g., the design of novel protein folds or in the search for high-affinity ligands.

Coulombic forces are modulated by the environment (Warshel et al., 2006), i.e., in case of soluble proteins by water and ions and possibly other proteins, and additionally by phospholipids in the case of membrane proteins. In atomistic molecular dynamics (MD) simulations (Karplus and McCammon, 2002), this environment is treated explicitly for a proper description, in particular, of the local electrostatics, e.g., in water-mediated hydrogen bonds. Computationally less demanding models introduced uniform dielectric constants for both the protein and the solvent (Tanford and Kirkwood, 1957), a distance-dependent dielectric constant accounting for electrostatic shielding within the solvent (Brooks et al., 1983), or modeled the solvent using an explicit grid of Langevin dipoles (Warshel and Levitt, 1976).

The electrostatic contribution to free energy differences between two states of a biomolecular system – e.g., two proteins bound to each other vs. the two proteins separated in space – is in many cases difficult to directly access; however, it may be computed from ensembles of microscopic structures. The simulation-based free energy perturbation (FEP) approach or the thermodynamic integration (TI) method is frequently used to analyze free energy difference of biomolecular states. The computational cost of such methods is, however, at variance with the need for high throughput, e.g., in protein design or protein–ligand docking and in the pK_a_ analysis of titratable sites as well. The latter problem requires the accurate estimation of the free energy differences between protonated and de-protonated states of all titratable groups in proteins, either on crystal structures or on trajectories obtained from molecular dynamics simulations to better grasp the protein flexibility (Narzi et al., 2008a). Protonation or pK changes are, e.g., important during the function of enzymes (Narzi et al., 2008a), or in protein–ligand binding (Narzi et al., 2008b; Onufriev and Alexov, 2013).

Due to the computational efficiency required to tackle the above problems, the solvation free energy is usually addressed in implicit models by solving the Poisson–Boltzmann equation (PBE) for the different states [see, e.g., Ullmann and Bombarda (2014)]. Different values for the dielectric continuum inside the protein have been used in the literature. The states may be crystal structures, modeled structures, snapshots from molecular dynamics simulations [MM/PBSA (Kollman et al., 2000)], or structural ensembles generated from crystal structures [CC/PBSA (Benedix et al., 2009)]. The solution of the PBE on structural ensembles should yield a more accurate solution for the solvation free energies of the studied biomolecules as it takes the flexibility of the protein into account.

A number of program packages for the numerical solution of the Poisson–Boltzmann equation have been developed in the past, namely APBS (Baker et al., 2001), UHBD (Madura et al., 1995), or DelPhi (Li et al., 2012), to mention a few. Here, we present a sequential PBE solver (*GroPBS*) that allows the accurate analysis of the electrostatic component of the solvation free energy of soluble proteins and of membrane proteins in explicit or implicit membranes. *GroPBS* is compatible with the GROMACS simulation suite and can, thus, easily be combined with simulations of biomolecules and the various biomolecular force fields available in Gromacs.

## 2 Methods

### 2.1 Poisson–Boltzmann Equation

**Figure 1:**
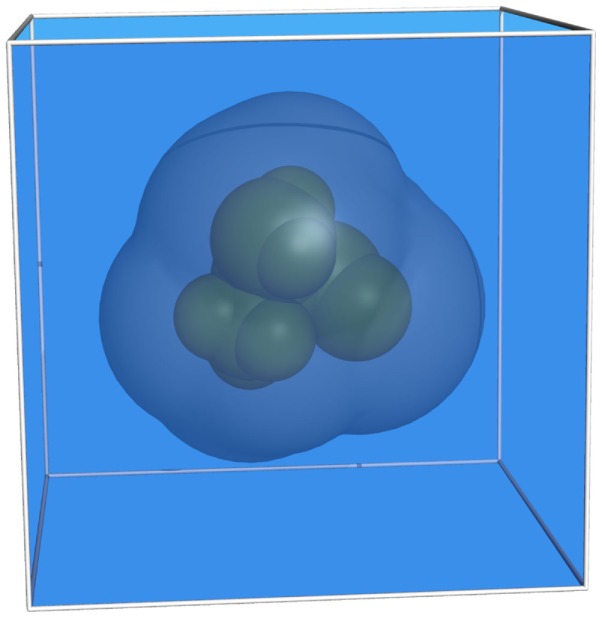
**Representation of a molecule consisting of nine atoms**. The different regions are colored as follows: green, van der Waals surface (inside); blue, solvent; dark blue, ion exclusion layer.

The analysis of the electrostatic potential Φ of a biomolecule in an implicitly treated solvent environment requires the numerical solution of the Poisson–Boltzmann equation (PBE). For the most simple case of a solute molecule in a homogeneous medium, three different domains (Holst, 1994) may be distinguished (see Figure 1):

1. Inside the molecule (green) (i.e., within the van der Waals radii of the atoms of the solute) Φ can be computed using the Poisson equation and Green’s identity resulting in
  $$\nabla^{2}\Phi \left(x\right)=\overset{M}{\underset{i=1}{\sum }}\frac{-4\pi q_{i}}{\varepsilon_{1}}\;\delta \left(x-x_{i}\right)$$
  where *M* is the number of atoms of the solute with point charges *q_i_* at positions *x_i_*.
2. Outside the molecule (light blue), the charge density of the ions in the solvent is assumed to follow a Boltzmann distribution that yields
  $$\nabla^{2}\Phi \left(x\right)=\kappa^{2}\frac{k_{B}T}{e_{c}}\sinh\left(\frac{e_{c}\Phi \left(x\right)}{k_{B}T}\right),$$
  *k_B_* is the Boltzmann constant, *e_c_* the elementary charge, *T* the temperature, and κ is the modified Debye–Hückel parameter, given by
  $$\kappa^{2}=\frac{8\pi N_{A}e_{c}^{2}I_{s}}{1000\varepsilon_{3}k_{B}T}$$
  with *N_A_* Avogadro’s number and *I_s_* the ionic strength of the solvent.
3. In the ion exclusion layer (dark blue) around the molecule no mobile ions are present. The Poisson equation reads accordingly
  $$\nabla^{2}\Phi \left(x\right)=0.$$

Combining these conditions into one equation, results in the non-linear PBE

(1) $$-\nabla \left(\varepsilon \left(x\right)\nabla \Phi \left(x\right)\right)+\kappa^{2}\left(x\right)\frac{k_{B}T}{e_{c}}\sinh\left(\frac{e_{c}\Phi \left(x\right)}{k_{B}T}\right)=4\pi \rho \left(x\right)$$

with the charge distribution function *ρ*(*x*) of the molecule and the spatial relative dielectric function ϵ(*x*) and κ^2^(*x*), which is 0 in the solute and the ion exclusion layer and κ^2^(*x*) = ϵ_3_κ^2^ for *x* in the solvent. ϵ(*x*) allows for the description of the dielectric discontinuity between the molecule and the solvent and is typically chosen to adopt values between 2 and 20 inside the biomolecule and 80 outside. More complicated models for the dielectric “constant” taking smoothed boundaries into account were suggested recently by Li et al. (2013) and will be considered for future work.

The framework provided by equation (1) allows the straightforward inclusion of various regions with different dielectric properties, e.g., of membranes. The membrane is modeled as another bulk medium like the solvent but with its own dielectric constant [ϵ_mem_ ≈ 2…3 (Böckmann et al., 2008)]. In a first step, the membrane is treated as a box in the *xy*-plane represented by its upper and lower *z*-value. However, more sophisticated models like curved membranes or taking the surface structure into account may easily be implemented.

The above partial differential equation cannot be solved analytically for objects shaped more complex than, e.g., a single sphere or cylinder. Therefore, the PBE has to be solved numerically.

### 2.2 Finite-Difference Method

The first step in the numerical solution of the PBE is to map all physical quantities (charges at atom centers, dielectric values, etc.) onto a three-dimensional uniform grid. Such a grid allows to replace differential operators by grid value differences. This approach is facilitated by linearizing the PBE [LPBE (Holst, 1994)]; For sinh(*x*) ≈ *x* the latter simplifies to

(2) $$-\nabla \left(\varepsilon \left(x\right)\nabla \Phi \left(x\right)\right)+\kappa^{2}\left(x\right)\Phi \left(x\right)=4\pi \rho \left(x\right).$$

Discretization of this equation yields for every grid point

(3) $$\Phi_{0}=\frac{\sum_{k=1}^{6}\varepsilon_{k}\Phi_{k}+4\pi q_{0}∕h}{\sum_{i=k}^{6}\varepsilon_{k}+{\left(\kappa_{0}\cdot h\right)}^{2}},$$

**Figure 2:**
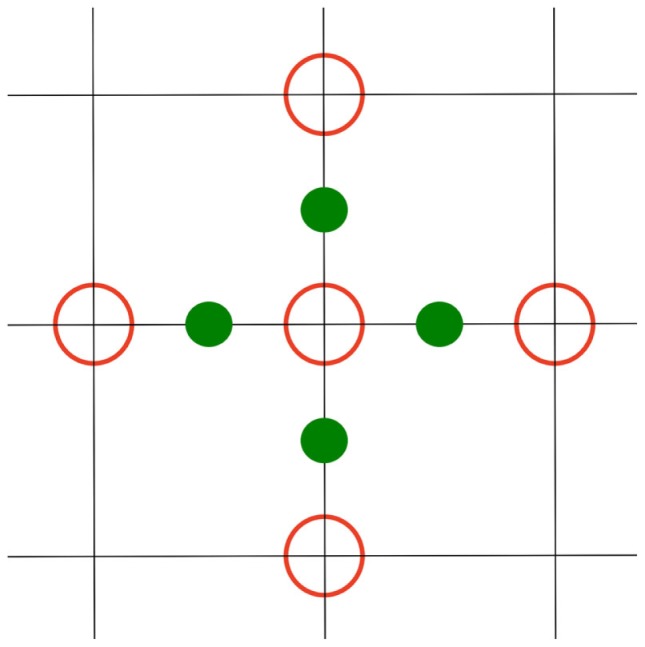
**2D representation of the computation scheme for equation (3): The inner red circle Φ_0_ is computed using the outer red circles Φ*_k_*, and the green circles ϵ_k_**.

*q*_0_ and κ_0_ denote the charge and Debye–Hückel parameter at the grid point, Φ*_k_* is the potentials at the six neighboring grid positions (in *x-*, *y-*, and *z*-direction), and ϵ*_k_* is the dielectric values at the midpoints between Φ_0_ and its neighbors Φ*_k_* (see Figure 2) (Nicholls and Honig, 1991). *h* is the step size, i.e., the distance between the grid points. While charge and the Debye–Hückel parameter are given values at the grid points, one important step is to define in which medium a grid point or midpoint is located. This problem is described in more detail below.

Application of equation (3) for each grid point results in a system of *N*^3^ linear equations where *N* is the grid size. This system can be reformulated as

(4) Φ=MΦ+v,

with Φ as a vector containing the potential at all grid points and *M* being a sparse matrix containing zeros at positions (*i*, *j*) if *i* and *j* are not neighboring grid points and $\frac{\varepsilon_{j}}{\sum_{k=1}^{6}\varepsilon_{k}+{\left(\kappa_{0}\cdot h\right)}^{2}}$ at positions (*i*, *j*) else, with *k* denoting all neighboring grid points of *i*. *v* is a vector containing the remaining terms of the discretized LPBE.

### 2.3 Successive Over-Relaxation

Several alternatives were suggested for the treatment of the grid boundaries: setting the electrostatic potential at the boundaries to 0, application of distance-dependent quasi-Coulombic boundary conditions, or periodic extensions of the system box in one or more directions.

The set of linear equations (4) is iteratively solved using methods like Jacobi or Gauss–Seidel (Demmel, 1997). Here, we used in a first serial implementation a successive over-relaxation (SOR) for Gauss–Seidel yielding the iteration rule

(5) $$\Phi^{\left(n+1\right)}=\omega \Phi^{\mathrm{new}}+\left(1-\omega \right)\Phi^{\left(n\right)},$$

*n* is the iteration step, Φ*^new^* is computed using equation (3). ω > 1 is the relaxation parameter. In a sequential implementation, every newly computed grid point value is immediately used for computing further grid points in the same iteration step – in contrast to the Jacobi method that renders the Gauss–Seidel approach much faster. Since the convergence rate of the Gauss–Seidel method depends on the spectral radius, i.e., the largest eigenvalue of *M*, ω should be chosen in a way it makes the spectral radius smaller. It can be shown that the optimal value is given by

$$\omega =\frac{2}{1+\sqrt{1-\lambda_{N}}},$$

λ*_N_* is the spectral radius. The spectral radius of *M* can be computed using the Connected-Moments Expansion (Cioslowski, 1987; Nicholls and Honig, 1991).

### 2.4 Solvation Free Energy

The grid-based electrostatic potential is used to compute the solvation free energy of the system. This is achieved by summing the product of the potential and the charge at each grid point, followed by subtraction of the corresponding energy as obtained for the potential using a uniform dielectric inside and outside of the solute. This approach eliminates the self-energy terms that are physically not meaningful. The disadvantage of this approach is the required double computation of the electrostatic potential.

A different approach can be used if the molecular surface is known (see below). The reaction field effects due to a dielectric boundary are replaced by the computation of the induced charges at this boundary (Rocchia et al., 2002). The solvation energy *G_W_* may then be calculated applying Coulomb’s law for the induced and the real charges. Formalizing this approach yields the following equation:

(6) $$\begin{array}{l} G_{W}=0.5\cdot \underset{b\in \mathrm{boundary}}{\sum }\left(\underset{p\in \mathrm{grid}}{\sum }\frac{q_{p}}{\mathrm{dist}\left(b_{s},p\right)}\right)\cdot \left(\frac{3h}{2\pi }\left(\Phi_{b}-\frac{1}{6}\overset{6}{\underset{k=1}{\sum }}\Phi_{k}\right)-q_{b}\right), \end{array}$$

*b* denotes the grid points at the dielectric boundary, *p* all grid points (these can be reduced to all charged grid points, as for all other points the term is 0) and *b_s_* is the points at the molecular surface obtained by projecting the boundary grid points to the surface.

### 2.5 Optimizations

Some simplifications of equation (3) are possible (Nicholls and Honig, 1991): first, most grid points do not hold a point charge, i.e., the term 4π*q*_0_/*h* is equal to 0. Second, most grid points are not found at a dielectric boundary, i.e., all neighboring midpoints are located in only one medium. These modifications lead to a quite simple 7-point stencil (modified by salt if present)

(7) $$\underset{\text{without salt}}{\underset{︸}{\Phi_{0}=\frac{1}{6}\overset{6}{\underset{k=1}{\sum }}\Phi_{k}}}\;\text{ or}\;\underset{\text{with salt}}{\underset{︸}{\Phi_{0}=\frac{\sum_{k=1}^{6}\Phi_{k}}{6+\left(\frac{{\left(\kappa_{0}\cdot h\right)}^{2}}{\varepsilon_{0}}\right)}}}.$$

In each iteration step of the SOR first this stencil is used, followed by analysis of the correction for dielectric discontinuities and charges if necessary. This approach is also a first step for a parallelization as the uniform stencil in equation (7) suits to be applied in parallel.

### 2.6 Extraction of Molecular Surface

In order to define which grid points lie inside and outside of the solute, the assignment of dielectric constants to the midpoints and, in particular for the computation of the free energy in a more sophisticated way (see above), the solute surface needs to be defined. The molecular surface is determined by its implicit description from the atom positions and radii of the solute. This surface is defined as the contact surface between the van der Waals surface and the surface of a spherical probe representing the solvent.

The first step is to determine a van der Waals map by mapping the atoms onto the grid. Every midpoint is categorized as inside or outside of the solute. This is done by examining all midpoints inside a box surrounding each atom and comparing their distance to the atom center with the radius of the atom. The same approach is used to determine which grid points are in solution, which is important for knowing which grid points’ stencil has to be modified by salt. Additionally, the grid points are classified as internal points if all surrounding midpoints are inside, external points if all surrounding midpoints are outside, or boundary points if some midpoints are in solution and some are not.

**Figure 3:**
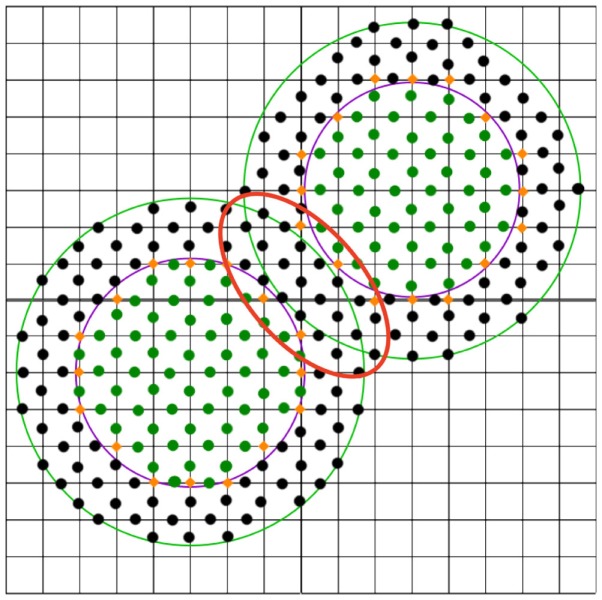
**2D representation of a very simple molecule consisting of two atoms on a grid**. The midpoints inside are marked as green dots, black dots (and no dots) denote midpoints outside. The grid points of interest at the boundary are marked with orange diamonds.

Taking into account the probe radius, some of these points have to be reclassified as sketched in the red marked region in Figure 3. This is done by iteratively examining the midpoints that surround the boundary points. For each such point, the distance to the solvent accessible surface is computed. This distance is defined as the distance from the van der Waals surface (violet line in Figure 3) extended by the radius of the probe, e.g., by 1.4 Å for water (one has to take into account that these extended atoms can overlap, see green line in Figure 3). If this distance is smaller than the probe radius, the midpoint remains outside; otherwise, it will be turned into an inside midpoint. Next, the grid points are reclassified using the new midpoints. This is repeated until no new boundary grid points are produced.

The actual surface points are finally constructed by projecting the boundary grid points onto the molecular surface either directly by moving them along the line connecting the grid point and the nearest atom center or by projecting them first onto the closest point of the solvent accessible surface and then back on the molecular surface in a similar way (Rocchia et al., 2002).

**Figure 4:**
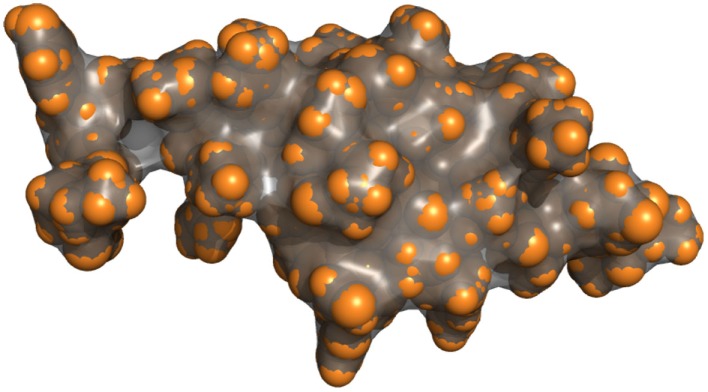
**Van der Waals surface of a peptide represented as spheres (orange) and its molecular surface (gray)**. The molecular surface and the van der Waals surface match for the convex parts of the molecule.

Figure 4 shows the result of the surface computation for a small peptide. In the convex parts, the molecular surface corresponds to the van der Waals surface (brightly colored parts), whereas they differ in concave parts and cavities.

## 3 Results

### 3.1 Evaluation of Current Program

The approach described in the Section “Methods” has been implemented in a sequential program using C++. As input separate files containing the atom positions (“.pdb,” Protein Data Bank format) and force field parameters, like in the program Delphi (Li et al., 2012) can be used as well as the Gromacs (Pronk et al., 2013) input file format (“.tpr”) for atom positions, Lennard Jones parameters for the atom sizes, and partial atomic charges. The leading biomolecular force fields for molecular modeling such as CHARMM, GROMOS, OPLS, or Amber are supported by Gromacs. This combination with the widely used Gromacs simulation package considerably simplifies the usage and enhances the applicability of the presented PBE solver in combination with biomolecular simulations, e.g., in the analysis of protein–protein binding free energies using the MM/PBSA approach.

The above-described input files and additional parameters like grid size, percentage of filling, probe radius, etc. are listed in one parameter file that serves as input. Table 1 contains all currently available parameters.

**Table 1:** **Possible parameters**.

in(tpr, path)	Gromacs input file	Can also be given in the command line
in(pdb, path)	Protein positions	Alternative to tpr
in(siz, path)	Protein sizes	Necessary with pdb, optional with tpr
in(crg, path)	Protein charges	Necessary with pdb, optional with tpr
in(sph, path)	Positions in pdb format	Optional, to compute the potential
		Along a path or a specific postilions

Filling	Percentage of box that is filled with protein	Default: 80%
Spacing	Spacing (in Å) between two grid points	Default: 1 Å
gridS	Grid size, i.e., number of grid points in each direction (odd)	Default: is computed
		Two of these three parameters
		Can be chosen

rmsc	Convergence criterion	Default: 0.0001
maxc	Convergence criterion	Default: 0.0001
maxit	Maximal iteration	Default: 500

epsIn	Dielectric constant inside the molecule	Default: 2.0
epsOut	Dielectric constant in the solution	Default: 80.0
salt	Concentration in moles/liter	Default: 0.0
temp	Temperature	Default: 273.15

bc = {1,2}	Boundary conditions	1: 0-boundary, 2: quasi-Coulombic
pb = *xyz*	Periodic boundary conditions in *x*-, *y*-, *z*-direction	Default: false

nomem	No membrane input	Default: no information
mem = zmin, zmax, eps	Minimal and maximal spread of membrane	Default: no information
	In *z*-direction and its dielectric constant	
tprmem = res, atm, eps	Residue and atoms to be considered membrane in tpr	Default: no information
	Its dielectric constant	

In the following, some of these parameters are explained in more detail.

#### 3.1.1 Membranes

Membranes are modeled as a slab in the *xy*-plane defined in extent by their minimal and maximal *z*-value. There are several possibilities to define the membrane extension:

- If the exact values are known beforehand, the mem = zmin,zmax,eps option in the parameter file can be used.
- These values can also be entered in the interactive mode after the *z*-extent of the molecule is reported.
- If a membrane is included in the “.tpr” file, with the tprmem = res,atm,eps option in the parameter file (or in the interactive mode) the associated residue names and atoms (e.g., POPC as residuum and P as atom) can be used to specify the membrane. The chosen atoms are separated into upper and lower parts of the membrane and the according center of mass is used as zmin and zmax.

#### 3.1.2 Boundary conditions

There are three possibilities to specify boundary conditions. These have to be fixed, as the grid points at the grid’s boundary do not have enough neighbors to be computed directly. The first and easiest way is to set the boundary grid points to 0. Another possibility is to approximate the potential at the boundary using quasi-Coulombic dipole conditions:

$$\Phi \left(x\right)=\frac{c_{+}\cdot \exp\left(\frac{-d_{+}}{\lambda }\right)}{d_{+}\cdot \varepsilon_{\mathrm{sol}}}+\frac{c_{-}\cdot \exp\left(\frac{-d_{-}}{\lambda }\right)}{d_{-}\cdot \varepsilon_{\mathrm{sol}}},$$

where ϵ*_sol_* is the dielectric constant of the solvent, *c*_+_ and *c*_−_are the total positive and negative charges, *d*_+_ and *d*_−_are the distance of the grid point to the center of the positive and negative charges, and λ denotes the Debye length.

Alternatively, periodic boundary conditions can be used in each grid dimension separately. Then for the missing neighbors, the corresponding points at the opposite side of the grid are used. This provides the opportunity to model infinite boxes.

#### 3.1.3 Convergence Criterion

There are three possible ways to define the convergence of the iterative procedure. The first is to set a threshold on the root mean squared change (rmsc), defined as

$$\mathrm{rms}c^{\left(i\right)}=\sqrt{\frac{1}{M}\underset{x}{\sum }{\left(\Phi^{\left(i\right)}\left(x\right)-\Phi^{\left(i-1\right)}\left(x\right)\right)}^{2}},$$

*M* is the number grid points and *i* is the iteration count. The rmsc measures the mean differences in the potential between two iterations. The option maxc limits the maximal change in the potential between two iterations. The third option allows to limit the number of iterations without regarding the convergence at all. Of course, these criteria can be combined, stopping the iteration process as soon as one criterion is fulfilled.

**Figure 5:**
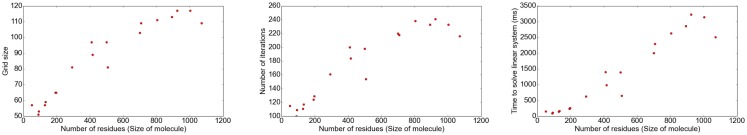
**The number of iterations and, therefore, the time to solve the linear system depends on the grid size, i.e., the size of the studied solute molecule**.

**Figure 6:**
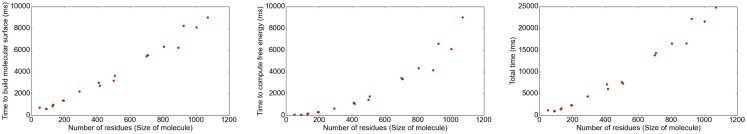
**The computation of the molecular surface, of the energy, as well as of the total CPU time depend on the number of residues**.

The implementation was tested using molecules of different sizes, ranging from small proteins with 49 residues to proteins as large as 1,070 residues (1,260–16,260 atoms, respectively). The grid size was chosen such that the filling rate was ≈80% using a grid size of 1 Å. Different results regarding the correlation between the number of atoms and grid size, number of iterations, and time consumption for different parts of the program are shown in Figures 5 and 6.

The results show that the number of iterations and, therefore, the time to solve the linear system strongly depends on the number of grid points. The surface computation and energy calculation, however, depend on the size of the molecule.

**Figure 7:**
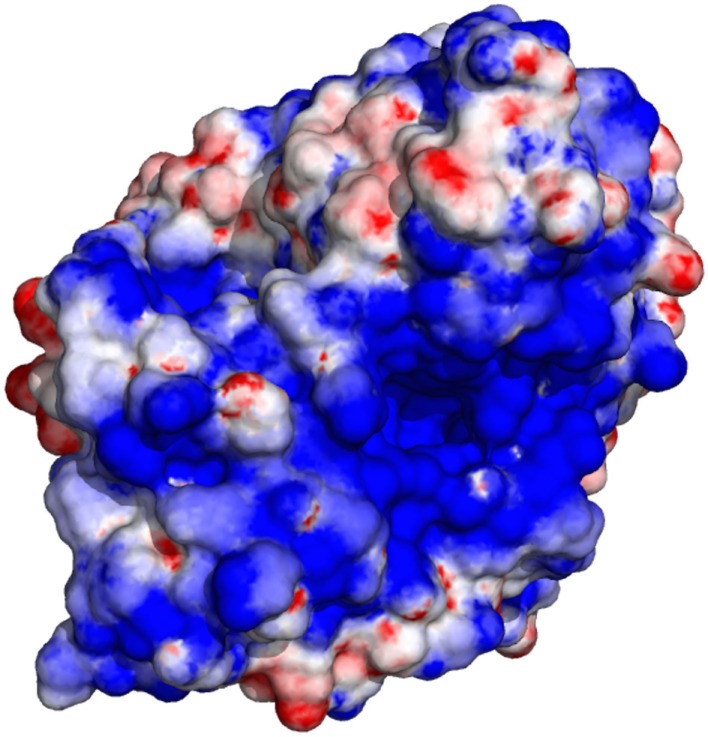
**Electrostatic surface potential of acetylcholinesterase (Colletier et al., 2006)**. Blue corresponds to an electrostatic potential of −4, red to +4 kT/e.

The electrostatic potential computed using GroPBS may be mapped onto the surface of biomolecules using, e.g., PyMOL (Schrödinger, 2010). As an example, Figure 7 shows the surface potential of acetylcholinesterase.

### 3.2 Influence of an Implicit Membrane

**Figure 8:**
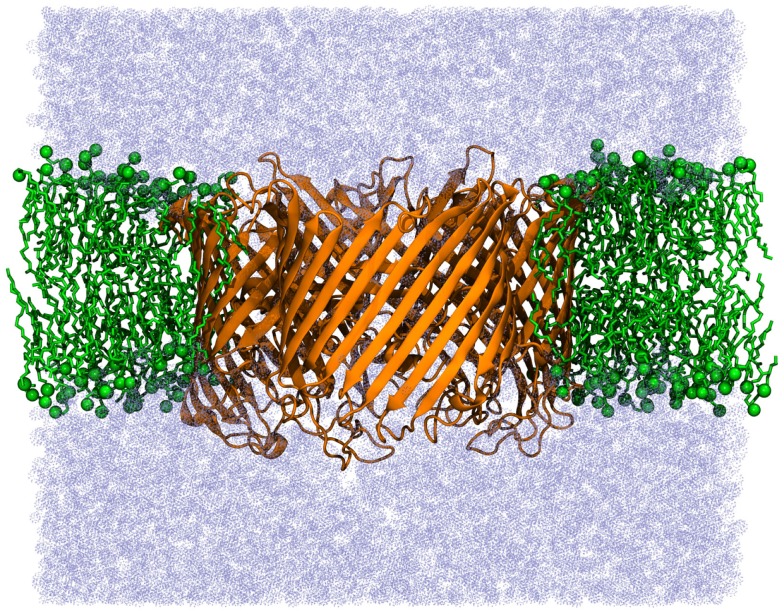
**Sucrose-specific porin ScrY embedded in a POPC membrane**.

In order to evaluate the influence of the low dielectric of a lipid membrane on the electrostatic potential of an embedded membrane protein, the potential was compared for the sucrose-specific porin ScrY (Forst et al., 1998) in different environments. Figure 8 shows this porin embedded in a POPC bilayer. The electrostatic potential was analyzed along a path through the pore of each monomer of the ScrY homo-trimer. This path was obtained using the program hole (Smart et al., 1993). For the solution of the PBE, the grid was chosen such that the filling rate was ≈80% using a step size of 0.5 Å and periodic boundary conditions in lateral direction (membrane plane) and dipole boundary conditions normal to the membrane. The dielectric constants were set to 4.0 inside the protein and 80.0 in the solvent phase. The dielectric of the surrounding implicit membrane was varied to study the influence of the membrane on the central, membrane-distal pore region. The PBE was solved every 0.5 ns of a 100 ns simulation, excluding the initial equilibration period of 20 ns. The electrostatic potential was averaged over the trajectory.

**Figure 9:**
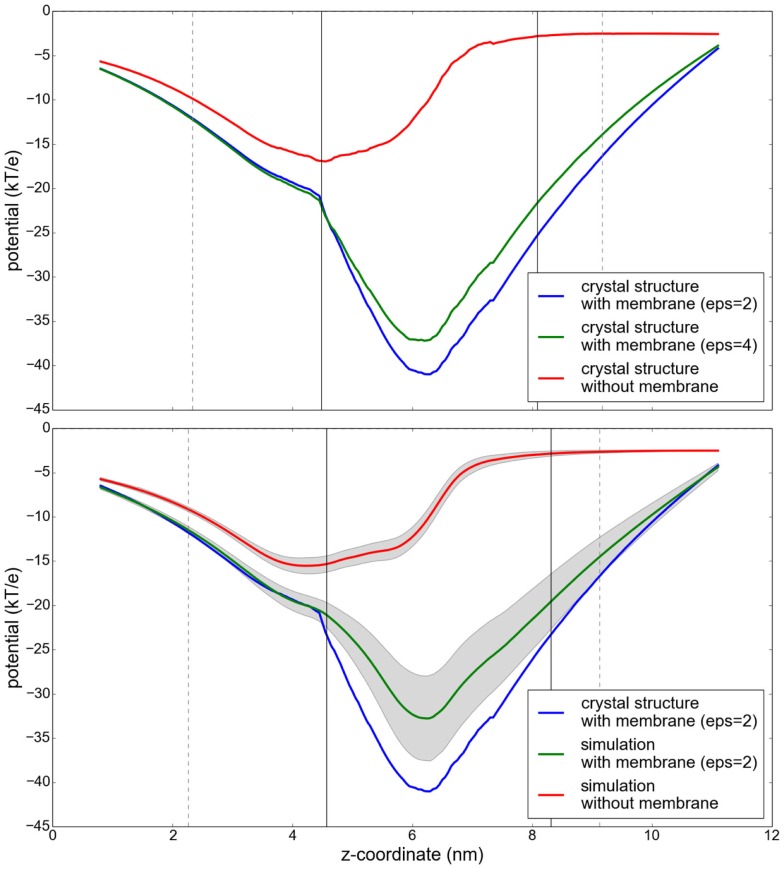
**Potential profiles along the paths through the sucrose-specific porin ScrY under different conditions**. Upper panel: the electrostatic potential computed without consideration of a membrane slab (red), and with an implicit membrane slab with ϵ = 2 (blue) and ϵ = 4 (green). Lower panel: Results for the electrostatic potential along the sucrose pore averaged over snapshots of a 100 ns molecular dynamics simulation. The protein flexibility is highlighted by its influence on the electrostatic potential (gray shaded region). The vertical lines describe the extent of the protein (gray) and the membrane (black).

The obtained electrostatic potential through the ScrY pore (see Figure 9, upper panel) is drastically decreased if an implicit membrane is included in the calculations. While the shape of the potential is similar, the minimum is shifted by approximately 1.5 nm, from the membrane interfacial region to the interior of the pore. The additional inclusion of flexibility by averaging the potential along a molecular dynamics trajectory results in an increase of the potential by up to 7 kT/e (Figure 9, lower panel).

## 4 Discussion and Future Work

A new program for the numerical solution of the Poisson–Boltzmann equation around biological macromolecules is presented (GroPBS). Apart from soluble proteins, GroPBS may as well be used to analyze the electrostatic potential of integral membrane proteins. The low-dielectric membrane environment may be modeled implicitly or explicitly. Additionally, GroPBS is shown to efficiently perform such computations both on pdb files and using the Gromacs input file format. This significantly simplifies the fast calculation of, e.g., the solvation free energies of biomolecules for different force fields or on ensembles of structures obtained from molecular dynamics simulations.

On the example of the sucrose-specific porin ScrY, we show that the inclusion of a membrane may have a substantial influence also on the potential inside the protein, and thus should not be neglected in PB calculations of membrane proteins.

In a subsequent step, GroPBS will be parallelized for multi-core architectures, and in particular, GPUs to enable, e.g., the fast analysis of changes in pK_a_ values on the fly during biomolecular simulations. Parallelization may be easily achieved by the so-called *checkerboard ordering* in the update of the electrostatic potential (Adams and Ortega, 1982).

The program will be made available free of charge on the following website: www.biotechnik.nat.uni-erlangen.de/research/boeckmann/downloads/GroPBS.

## Author Contributions

Research was designed by FB and RB, performed by FB and LS, and the manuscript was written by all.

## Conflict of Interest Statement

The authors declare that the research was conducted in the absence of any commercial or financial relationships that could be construed as a potential conflict of interest.
